# microRNA and gene networks in human pancreatic cancer

**DOI:** 10.3892/ol.2013.1521

**Published:** 2013-08-09

**Authors:** MINGHUI ZHU, ZHIWEN XU, KUNHAO WANG, NING WANG, YANG LI

**Affiliations:** 1 College of Computer Science and Technology, Jilin University, Changchun, Jilin 130012, P.R. China; 2 Key Laboratory of Symbolic Computation and Knowledge Engineering of the Ministry of Education, Jilin University, Changchun, Jilin 130012, P.R. China

**Keywords:** pancreatic cancer, microRNA, transcription factor, network, pathway, host gene

## Abstract

To date, scientists have obtained a substantial amount of knowledge with regard to genes and microRNAs (miRNAs) in pancreatic cancer (PC). However, deciphering the regulatory mechanism of these genes and miRNAs remains difficult. In the present study, three regulatory networks consisting of a differentially-expressed network, a related network and a global network, were constructed in order to identify the mechanisms and certain key miRNA and gene pathways in PC. The interactions between transcription factors (TFs) and miRNAs, miRNAs and target genes and an miRNA and its host gene were investigated. The present study compared and analyzed the similarities and differences between the three networks in order to distinguish the key pathways. Certain pathways involving the differentially-expressed genes and miRNAs demonstrated specific features. TP53 and hsa-miR-125b were observed to form a self-adaptation association. A further 16 significant differentially-expressed miRNAs were obtained and it was observed that an miRNA and its host gene exhibit specific features in PC, for example, hsa-miR-196a-1 and its host gene, HOXB7, form a self-adaptation association. The differentially-expressed network partially illuminated the mechanism of PC. The present study provides comprehensive data that is associated with PC and may aid future studies in obtaining pertinent data results with regards to PC. In the future, an improved understanding of PC may be obtained through an increased knowledge of the occurrence, mechanism, improvement, metastasis and treatment of the disease.

## Introduction

Pancreatic cancer (PC) is the fourth primary cause of cancer-related mortality and <5% of patients survive for five years following the diagnosis, with a median survival time of 4–6 months (1).

Transcription factors (TFs) and microRNAs (miRNAs) are prominent regulators for gene expression (2). TFs are proteins that are able to activate or repress transcription by binding to *cis*-regulatory elements that are located in the upstream regions of genes. miRNAs are small (21–24 nt) non-coding RNA molecules that control key cellular processes, including proliferation, differentiation and apoptosis. Zhang *et al* demonstrated that miRNAs were identified to play fundamentally important roles in PC (3). The study illustrated the biogenesis and differential expression of miRNAs and their corresponding potential roles in the pathogenesis, progression and metastasis of PC.

Several transcription profiling studies involving miRNA transfection experiments have shown that miRNAs exert a widespread impact on the regulation of their target genes (targets) (4). To date, numerous databases supply abundant resources to study the association between miRNAs and their targets.

Host genes are those genes to which the miRNAs locate. Rodriguez *et al* indicated that miRNAs are transcribed in parallel with their host transcripts and identified two transcription classes of miRNAs, exonic and intronic (5). Baskerville *et al* identified that intronic miRNAs are closely associated with their host genes (6).

Numerous differentially-expressed genes and miRNAs have been identified through a large quantity of PC experiments (7). However, the experiments are mostly based on a single element (gene or miRNA), which makes it difficult to analyze the general pathogenesis of PC. The present study focused on the underlying network of miRNAs, the targets of the miRNAs, the TFs and the host genes of the miRNAs in order to aid in the identification of the key pathway control mechanisms of PC.

## Materials and methods

### Material collection and data processing

The experimentally validated dataset of human miRNAs and their targets were extracted from Tarbase 5.0 (8) and miRTarBase (9) and were considered as set *A_1_*.

The experimentally validated dataset of human TFs and the miRNAs that were regulated by them were extracted from TransmiR (10) and were considered as set *A_2_*.

The host genes of the human miRNAs were extracted from miRBase (11) and the National Centre for Biotechnology Information (NCBI). Official symbols and IDs were used to signify each host gene. This data set was considered as *A_3_*.

The differentially-expressed genes were collected from the Kyoto Encyclopedia of Genes and Genomes (KEGG) pathway database (12), the Cancer Genetics Web (http://www.cancerindex.org/geneweb/index.htm), the NCBI SNP database (http://www.ncbi.nlm.nih.gov/snp/) and from the relevant literature. The PC-related genes were collected from the GeneCards database (13) and relevant literature, and included the genes that affect tumor growth, migration, radial therapy and the clinical outcome of patients with PC. Additionally, 22 popular TFs were extracted using the P-match method (14) and were considered as PC-related genes. The present study only focused on the TFs that appeared in transmiR. Promoter region sequences of 1,000 nt in length that are targeted by differentially-expressed miRNAs were downloaded from the University of California Santa Cruz (UCSC) database (15). The P-match method, which combines pattern matching and weight matrix approaches, was used to identify the TF-binding sites (TFBSs) in the 1,000-nt promoter region sequences, which were mapped onto the promoter region of the targets. The matrix library of P-match contains sets of known TFBSs that have been collected in TRANSFAC, allowing the possibility of searching for a large variety of TFBSs. The vertebrate matrix was used with a restricted high quality criterion, which was considered as set *A_4_*.

Differentially-expressed miRNAs were collected from mir2Disease (16) and the relevant literature was examined to extract PC-related miRNAs. This dataset was considered as set *A_5_*.

### Network construction

The following method was used to construct the differentially-expressed, related and global networks. All the regulatory factors for the TFs, miRNAs, targets and host genes were extracted from datasets *A_1_*, *A_2_* and *A_3_*. The global network was obtained as a result of combining the regulatory factors. The differentially-expressed elements were separately extracted from datasets *A_4_* and *A_5_*, and the factors with differentially-expressed elements were selected from the global network, thus forming the differentially-expressed network. A similar method was used to construct the related network.

## Results

### Differentially-expressed PC network

**Figure 1:**
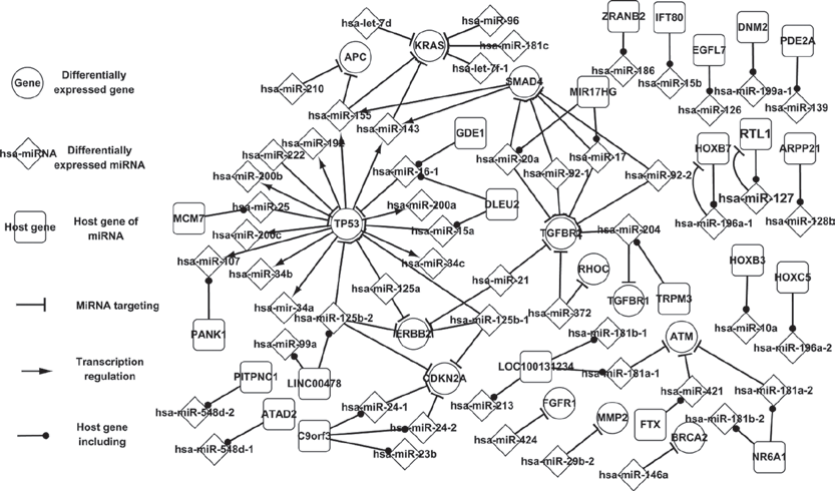
Differentially-expressed network of the genes and miRNAs in PC. All elements are involved in various progressions of PC. TP53 participates in the cell cycle and hsa-miR-10a contributes to the proliferation of PC cells. The associations between the differentially-expressed genes and miRNAs are shown in this network, which partly reveal the mechanism of PC. miRNA, microRNA; PC, pancreatic cancer.

Fig. 1 shows the significant regulatory factors of the differentially-expressed elements in PC. This network is composed of three TFs (TP53, SMAD4 and CDKN2A), targets of miRNAs, miRNAs and host genes. The nodes are all differentially expressed with the exception of the host genes. The three TF-related pathways are the most significant among them. hsa-miR-17 targets SMAD4, which regulates hsa-miR-143 and hsa-miR-155. Navarro *et al*(17) revealed that TP53 regulates hsa-miR-143. Xu *et al*(18) identified that hsa-miR-143 targets KRAS. TP53 has been suggested to indirectly affect KRAS by hsa-miR-143. Zhang *et al*(19) indicated that hsa-miR-155 targets APC. Therefore, it was concluded that SMAD4 was able to indirectly affect APC by hsa-miR-155. TP53 directly regulates hsa-miR-125b and in turn, hsa-miR-125b targets TP53 (20), suggesting a self-adaptation association between the two. Certain miRNA pathways and the corresponding host genes are highlighted in the present study. RTL1 and hsa-miR-127, and HOXB7 and hsa-miR-196a-1 form self-adaptation associations. A host gene may include several miRNAs. C9orf3 contains hsa-miR-24 and hsa-miR-23b. Furthermore, an miRNA may be located in several genes. hsa-miR-548d is present in ATAD2 and PITPNC1. The combined action of the nodes in the differentially-expressed network partially revealed the regulatory mechanism of PC.

### Related PC network

**Figure 2:**
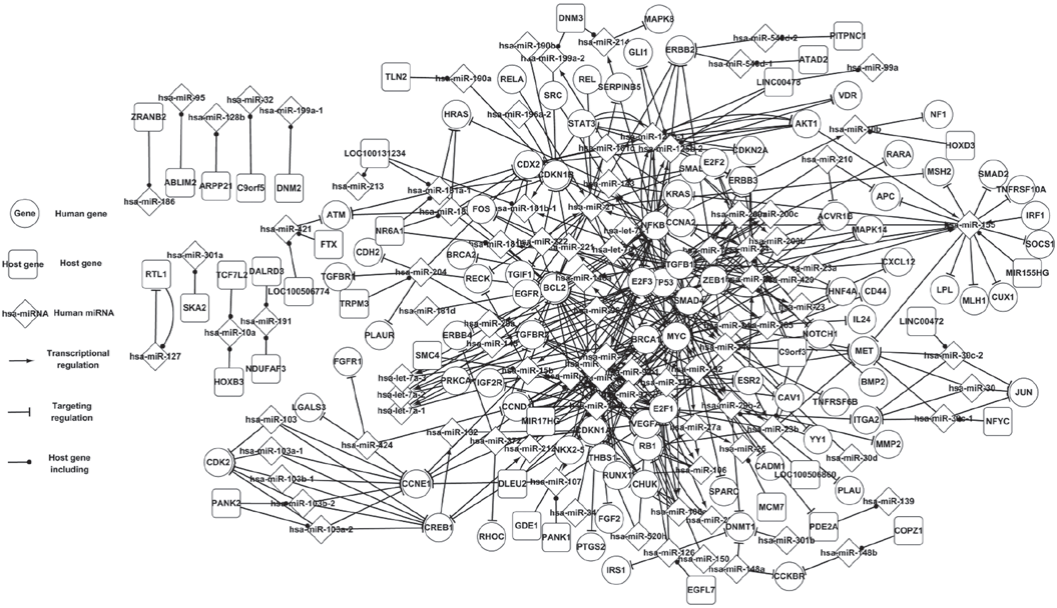
Related network of the genes and miRNAs in PC showing seven related miRNAs and additional pathways. The related network expands on the additional topological associations of the differentially-expressed elements. miRNA, microRNA; PC, pancreatic cancer.

Fig. 2 shows the numerous regulatory associations in PC with regard to genes and miRNAs. The related network incorporates the differentially-expressed network. Fig. 2 shows three differentially-expressed TFs and 23 additional TFs. With the exception of the differentially-expressed miRNAs, seven related miRNAs (hsa-miR-145, hsa-miR-29a, hsa-miR-30d, hsa-let-7a-1, hsa-let-7a-2, hsa-let-7a-3 and hsa-let-7i) are shown. Fig. 2 also depicts additional gene and miRNA pathways. MYC and ZEB1 regulate hsa-let-7d, which targets KRAS. AKT1, NFKB1, STAT3 and CDX2 regulate hsa-miR-125b, which in turn regulates TP53, AKT1 and STAT3. Qiu *et al*(21) elucidated that the activation of the STAT3 signaling pathway plays a significant role in the progression of PC. The related network expands the additional topological associations of the differentially-expressed elements and contributes to a further understanding of the pathogenesis of PC.

### Global PC network

The global network involves more comprehensive regulatory associations, including *A_1_*, *A_2_* and *A_3_*, and is an experimentally validated biological network in the human body. The network incorporates the differentially-expressed and related networks.

### Host genes and miRNAs in PC

**Figure 3:**
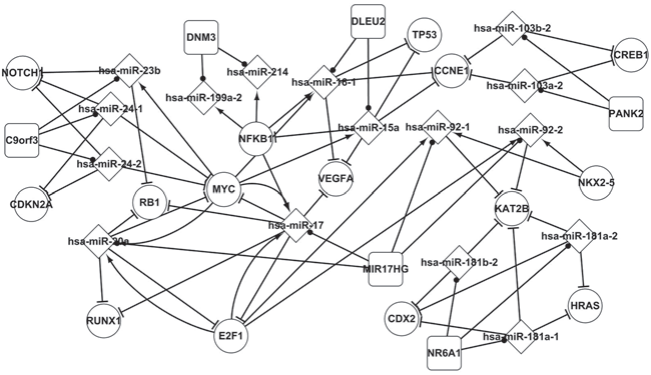
Significant associations between the host genes and their miRNAs in the PC-related network. miRNAs and their host genes are able to affect the progression of certain cancers. A host gene may be associated with several miRNAs that together target the same genes or individually target certain genes. miRNA, microRNA; PC, pancreatic cancer.

Fig. 3 shows certain significant characteristics of the host genes and their miRNAs. Although the host genes themselves are not differentially expressed in PC, they are considered to be so since their miRNAs are differentially expressed. DLEU2 contains hsa-miR-15a and hsa-miR-16-1, which are regulated by MYC and together target VEGFA, TP53 and CCNE1. MIR17HG is a host gene for hsa-miR-92-1, hsa-miR-92-2, hsa-miR-17 and hsa-miR-20a, which are regulated by E2F1. hsa-miR-17 and hsa-miR-20a separately form self-adaptation associations with MYC and E2F1. PANK2 includes hsa-miR-103a-2 and hsa-miR-103b-2, which target CCNE1 and CREB1.

### Transcriptional network of popular TFs

**Figure 4:**
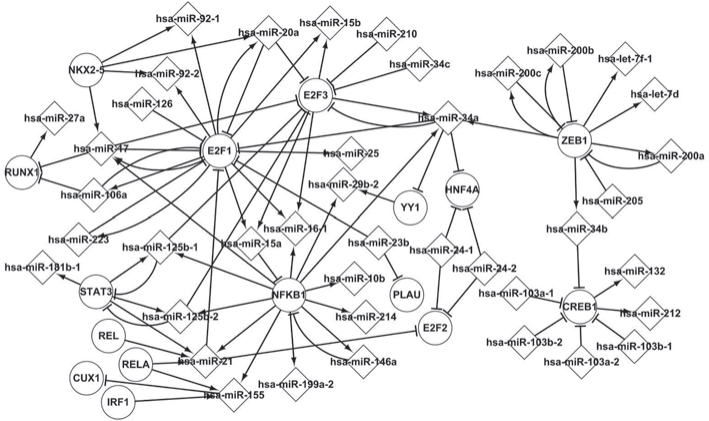
Transcription network of popular TFs and differentially-expressed miRNAs and the genes in PC showing the popular TFs that regulate differentially-expressed miRNAs, which target differentially-expressed genes. These popular TFs are frequently involved in the transcriptional progression of cancer. TF, transcription factor; miRNA, microRNA; PC, pancreatic cancer.

A further 41 differentially-expressed miRNAs that are regulated by popular TFs were analyzed. Fig. 4 shows the regulatory interactions between these popular TFs and the differentially-expressed miRNAs, in addition to the targets in PC. The TFs and miRNAs affect their successors in the transcriptional network. A total of four TFs, NFKB1, ZEB1, E2F1 and E2F3, have been experimentally validated in PC. Fig. 4 shows the TFs, ZEB1, E2F3 and NFKB1, which coregulate hsa-miR-34a, which in turn targets E2F3, YY1, HNF4A and E2F1. Fig. 4 also shows that one differentially-expressed miRNA may be regulated by several TFs, that a target is targeted by several differentially-expressed miRNAs and that a TF indirectly affects other genes by several differentially-expressed miRNAs. hsa-miR-16-1 is regulated by E2F1, E2F3 and NFKB1. RUNX1 is targeted by hsa-miR-17 and hsa-miR-106a. ZEB1 indirectly regulates CREB1 by hsa-miR-34b, and hsa-miR-125b indirectly affects hsa-miR-21 by STAT3. The transcriptional network of popular TFs and miRNAs may aid in the analysis of the pathogenesis of PC.

### Regulatory pathway of differentially-expressed genes

The upstream and downstream information of the differentially-expressed genes and miRNAs and the popular TFs was extracted in order to describe the PC network more clearly.

The successor and precursor nodes of the differentially-expressed genes in the three networks were extracted to compare and analyze the regulatory pathway. Among these genes, TP53 and SMAD4 were observed to exhibit a characteristic that was common to the precursor and successor nodes, indicating that the gene and miRNA form a self-adaptation association.

Using TP53 as an example, Table I shows TP53 and the predecessors and successors of the gene, in addition to their regulatory associations. TP53 is a notable tumor suppressor and is significantly featured in the three networks. Table I shows eight miRNAs that target TP53, which itself regulates 12 miRNAs in the differentially-expressed network. Therefore, the eight miRNAs indirectly affect the expression of the 12 miRNAs through TP53. hsa-miR-125b and TP53 were observed to regulate each other in the three networks, indicating that they are crucial in the progression of PC. A mutation in the TP53 gene is implicated in the pathogenesis of PC through the constitutive activation of the p53 pathway.

### Regulatory pathway of differentially-expressed miRNAs

Similar to the differentially-expressed genes, the differentially-expressed miRNA pathways were extracted, compared and analyzed using the same method. Among these miRNAs, 13 differentially-expressed miRNAs (hsa-miR-146a, hsa-miR-34a, hsa-miR-125b-1, hsa-miR-125b-2, hsa-miR-17-5p, hsa-miR-200a, hsa-miR-200b, hsa-miR-200c, hsa-miR-20a, hsa-miR-21, hsa-miR-223, hsa-miR-24-1 and hsa-miR-429) and their corresponding genes were observed to form self-adaptation associations.

Using hsa-miR-125b as an example, Table II presents hsa-miR-125b and the predecessors and successors of the miRNA, in addition to the regulatory associations between them. Table II indicates that TP53 regulates hsa-miR-125b that target TP53, CDKN2A and ERBB2 in the differentially expressed network. Certain targets of the global network have been omitted. Table II also shows that AKT1 and hsa-miR-125b, and STAT3 and hsa-miR-125b separately form self-adaptation associations. hsa-miR-125b also indirectly affects other miRNAs by certain TFs. hsa-miR-125b targets TP53, which regulates hsa-miR-143 and hsa-miR-155. Certain miRNAs also indirectly affect hsa-miR-125b through TFs. hsa-miR-15a targets NFKB1, which regulates hsa-miR-125b.

### Regulatory pathway of popular TFs

The same method was used to extract, compare and analyze the pathways of each popular TF in the related network. A total of five TFs, E2F1, E2F3, NFKB1, STAT3 and ZEB1, which are associated with PC, and their corresponding miRNAs were observed to form self-adaptation associations.

Using E2F1 as an example, Table III shows that four differentially-expressed miRNAs, hsa-miR-106a, hsa-miR-17, hsa-miR-20a and hsa-miR-223, separately form self-adaptation associations with E2F1. E2F1 is not differentially expressed in PC, but the four miRNAs are differentially expressed. Therefore, the four miRNAs may indirectly lead to the aberrant expression of other miRNAs by E2F1. The E2F1 and differentially-expressed miRNA pathways indicated an additional 14 significant differentially-expressed miRNAs in PC. These miRNAs are not only differentially expressed, but are also adjacent nodes of E2F1 that are frequently involved in the transcription of cancer.

## Discussion

The present study derived three regulatory networks, differentially-expressed, related and global, by analyzing the current experimentally validated genes and miRNAs that are associated with PC. The results revealed certain significant pathways in PC and identified a topological network of the development of PC. Certain pathways possessed specific features; 13 differentially-expressed miRNAs and corresponding genes were shown to form self-adaptation associations. Self-adaptation associations were also identified in five popular TFs and 11 differentially-expressed miRNAs. A further 41 significant differentially-expressed miRNAs were obtained by comparing the differentially-expressed and popular transcription networks. hsa-miR-127 and its host gene, RTL1, and hsa-miR-196a-1 and its host gene, HOXB7, were observed to separately form self-adaptation associations. Certain pathways have not only been found in PC, but also in other carcinomas. For example, TP53 drives invasion in breast tumors through the upregulation of hsa-miR-155. TFBSs were identified by combining pattern matching and weight matrix approaches in the 1,000-nt promoter region sequences, and were then mapped onto the promoter region of the targets. The TFs predicted from this method suggest the potential correlations between the differentially expressed miRNAs and TFs. More attention should be paid to them and experiments should be conducted to validate the close correlations with PC. The present study partially uncovered the regulatory associations in the development of PC and supplied comprehensive data associated with PC. Certain key pathways may aid studies to further investigate the carcinogenicity mechanism and therapy of PC. In future studies, the interaction of proteins and regulatory patterns, including upregulation and downregulation, will be taken into account and a more comprehensive and extensive network of PC will be constructed. As a consequence of these studies, the prognosis, diagnosis and therapy of PC may be improved.

## Figures and Tables

**Table I:** Regulatory association between miRNAs and TP53.

miRNAs that target TP53				miRNAs that are regulated by TP53		
		
Differentially-expressed network	Related network	Global network	Gene symbol	Differentially-expressed network	Related network	Global network
miR-125a	miR-125a	miR-125a	TP53	miR-107	miR-107	miR-107
miR-125b-1	miR-125b-1	miR-125b-1		miR-125b-1	miR-125b-1	miR-125b-1
miR-125b-2	miR-125b-2	miR-125b-2		miR-125b-2	miR-125b-2	miR-125b-2
miR-15a	miR-15a	miR-15a		miR-143	miR-143	miR-143
miR-16-1	miR-16-1	miR-16-1		miR-155	miR-155	miR-155
miR-221	miR-221	miR-221		miR-192	miR-192	miR-192
miR-222	miR-222	miR-222		miR-200a	miR-200a	miR-200a
miR-25	miR-25	miR-25		miR-200b	miR-200b	miR-200b
	miR-30d	miR-30d		miR-200c	miR-200c	miR-200c
		miR-612		miR-34a	miR-34a	miR-34a
				miR-34b	miR-34b	miR-34b
				miR-34c	miR-34c	miR-34c
					miR-145	miR-145
					miR-29a	miR-29a
						miR-215
						miR-29b-1
						miR-29b-2
						miR-29c
						miR-194-1
						miR-194-2
						miR-519d

miRNA, microRNA.

**Table II:** Regulatory associations between hsa-miR-125b and genes.

Genes that regulate hsa-miR-125b				Genes that are targeted by hsa-miR-125b		
		
Differentially-expressed network	Related network	Global network	miRNA symbol	Differentially-expressed network	Related network	Global network
TP53	TP53	TP53	miR-125b	TP53	TP53	TP53
	AKT1	AKT1		CDKN2A	CDKN2A	CDKN2A
	NFKB1	NFKB1		ERBB2	ERBB2	ERBB2
	STAT3	STAT3			AKT1	AKT1
	CDX2	CDX2			STAT3	STAT3
					BBC3	BBC3
					E2F3	E2F3
					ERBB3	ERBB3
					GLI1	GLI1
					IGF2	IGF2
					VDR	VDR
						ABCC4
						ATXN1
						BMF

miRNA, microRNA.

**Table III:** Regulatory associations between miRNAs and E2F1.

miRNAs that target E2F1				miRNAs that are regulated by E2F1		
		
Differentially-expressed network	Related network	Global network	Gene symbol	Differentially-expressed network	Related network	Global network
miR-106a	miR-106a	miR-106a	E2F1	miR-106a	miR-106a	miR-106a
miR-126	miR-126	miR-126		miR-15a	miR-15a	miR-15a
miR-17	miR-17	miR-17		miR-15b	miR-15b	miR-15b
miR-20a	miR-20a	miR-20a		miR-16-1	miR-16-1	miR-16-1
miR-21	miR-21	miR-21		miR-17	miR-17	miR-17
miR-223	miR-223	miR-223		miR-20a	miR-20a	miR-20a
miR-23b	miR-23b	miR-23b		miR-223	miR-223	miR-223
miR-34a	miR-23b	miR-34a		miR-25	miR-25	miR-25
	miR-23b	let-7a-1		miR-92-1	miR-92-1	miR-92-1
	let-7a-1	let-7a-2		miR-92-2	miR-92-2	miR-92-2
	let-7a-2	let-7a-3			let-7i	miR-92-2
	let-7a-3	miR-149				let-7i
		miR-106b				let-7a-1
		miR-330				let-7a-2
		miR-93				let-7a-3
		miR-98				miR-195
						miR-19a
						miR-106b
						miR-16-2
						miR-18a
						miR-18b
						miR-19b-1
						miR-19b-2
						miR-20b
						miR-363
						miR-449a
						miR-449b
						miR-449c
						miR-93

miRNA, microRNA.

## Abbreviations

miRNA: microRNA
TFs: transcription factors
targets: target genes
PC: pancreatic cancer
NCBI: National Center for Biotechnology Information
TFBSs: transcription factor binding sites
